# Metropolitan Hospital Sunday Fund

**Published:** 1906-12-08

**Authors:** 


### METROPOLITAN HOSPITAL SUNDAY FUND.

### TRIBUTES TO THE LATE SIR SYDNEY WATERLOW AND GEORGE HERRING.

At a meeting of the Council of the Metropolitan Hospital Sunday Fund, held at the Mansion House on Thursday, November 29, the following tributes were paid to

#### The Late George Herring.

The Earl of Stamford said he felt it had been a privilege to have found himself in constant association with the late Mr. Herring in the work of the Distribution Committee. Mr. Herring's character must have impressed everyone with whom he came in contact by his rugged sincerity and his extraordinary ability in this as in other business matters with which he was connected. It was remarkable how he always seemed to get right, and to go straight to the point. There was something very attractive about his moral character. Always genial and patient, he was ready to hear the other side, and to take it with perfect good humour when he was overborne in debate. The Committee of Distribution was a very harmonious body, but occasionally differences of opinion arose, and he had sometimes found himself in a minority of two with George Herring, who was always prepared to acquiesce in the decision of the majority with perfect good temper. A very notable thing about him was the wonderful absence of self-seeking. He might have had a good deal of distinction at his command had he chosen to seek for it, but he seemed only to regard the welfare of his fellow-men. Only the day before the operation which preceded his death he was sitting next to Mr. Herring at one of their meetings; and though he was desirous of getting away, yet when he (the speaker) put before him a pressing matter he had very much at heart, he could not help being struck again by the geniality and patience with which Mr. Herring listened, the ability with which he grasped the whole thing, and his readiness to give assistance. It was with mingled feelings he moved the resolution committed to him, namely:—

That the Council has heard with great regret of the death of its esteemed colleague, Mr. George Herring, who was a regular attendant at the meetings of the Distribution Committee, and whose munificent gifts, extending over many years, assisted in largely increasing the amount of the annual collections.

Sir William Church seconded, and said he should like to bear testimony to the extreme regard which all members of the Distribution Committee felt for Mr. George Herring. When he joined it was not long before they realised that they had a great increase of strength, and of this they were conscious throughout his connection with them.

#### The Late Sir Sydney Waterlow.

Speaking to the following resolution:

That this Committee desire to offer their sincere condolence with Lady Waterlow on the death of Sir Sydney Waterlow, Bart., who, since the first inauguration of the Hospital Sunday Fund, had been one of its most prominent workers and warmest friends,

Canon Fleming said no friend of Sir Sydney Waterlow could feel it a greater privilege than he did to move that resolution, and in reading it he felt that it reviewed the whole history of the Hospital Sunday Fund. That Fund owed its origin to the suggestion of an old friend of his, the Rev. Canon Miller, who, when Rector of Birmingham, where he won his spurs by the work he did for that remarkable city, started the idea of a simultaneous collection for the hospitals from all churches and all creeds. When Mr. Gladstone preferred him to be Vicar of Greenwich, one of the first he named it to was Sir Sydney Waterlow, who, when he had given the scheme due consideration, laid hold of it with head and heart and hands. Within a month of his election to the Mayoralty, on December 11, 1872, Sir Sydney called the first meeting to deal with it; and in his year of office the first Hospital Sunday was carried out—in June 1873. Those who knew the difficulties of bringing together varying creeds and denominations would realise the indomitable energy which Sir Sydney threw into the work when it was recalled that to that first collection 1,072 congregations contributed, and that the virgin result was £27,700. They knew well that the first year of any great work was its most difficult year, for a mistake then might mar everything. But Sir Sydney was not one to make many mistakes. His remarkable character, his hunger for work and his capacity for doing it ensured success for whatever he took up. This Fund was no exception. The continuity of its success, which had this year reached 2,013 congregations and £63,047, must be traced to his inauguration and to the fact that every Lord Mayor of London, since its initiation, had felt it a privilege and an honour to give it the prestige which his high office conferred upon all good works within the walls of the Mansion House. They all mourned Sir Sydney Waterlow that day, and from their hearts desired to accord this resolution of condolence with Lady Waterlow. They were the inheritors of the work which he nursed, tended, and built up to the last. When he thought of the friend whom they all revered and honoured and many of them loved, he recalled the words of Robert Browning. He was—

> One who never turned his back but marched breast forward,
> Never doubted clouds would break,
> Never dreamed, though right were worsted, wrong would triumph,
> Held we fall to rise, are baffled to fight better,
> Sleep to wake.

We refer to this meeting and to the origin of the Hospital Sunday Fund in London under Current Hospital Topics on page 181.

